# Uncertainty Quantification of the Mechanical Properties of 2D Hexagonal Cellular Solid by Analytical and Finite Element Method Approach

**DOI:** 10.3390/ma18204792

**Published:** 2025-10-20

**Authors:** Safdar Iqbal, Marcin Kamiński

**Affiliations:** Department of Structural Mechanics, Faculty of Civil Engineering, Architecture and Environmental Engineering, Lodz University of Technology, 93-590 Lodz, Poland; safdar.iqbal@dokt.p.lodz.pl

**Keywords:** hexagonal unit cell, uncertainty quantification, homogenization method, finite element method, Monte Carlo simulation, stochastic perturbation method, relative entropy

## Abstract

The mechanical properties of cellular materials are critical to their performance and must be accurately determined through both analytical and numerical methods. These approaches are essential not only for understanding material behavior but also for evaluating the effects of parametric variations within the unit cell structure. This study focuses on the in-plane comparison of analytical and numerical evaluations of key mechanical properties, including Young’s modulus, yield strength, and Poisson’s ratio of a 2D hexagonal unit cell subjected to systematic geometric and material variations. Analytically, the mechanical properties were derived based on the geometric configuration of the hexagonal unit cell. Numerically, the finite element method (FEM) simulations employed three different meshing methods: quadrilateral, quad-dominated, and triangular elements, to ensure precision and consistency in the results. The elastic response (Young’s modulus) was examined through a parametric sweep involving segmental length variations (4.41 to 4.71 mm) and material modulus (66.5 to 71.5 GPa), revealing percentage differences between analytical and numerical results ranging from −8.28% to 10.87% and −10.58% to 11.95%, respectively. Similarly, yield strength was evaluated with respect to variations in segmental length (4.41 to 4.71 mm) and wall thickness (1.08 to 1.11 mm), showing discrepancies between −2.86% to −5.53% for segmental length and 7.76% to 10.57% for thickness. For Poisson’s ratio, variations in the same parameters led to differences ranging from −7.05% to −12.48% and −9.11% to −12.64%, respectively. Additionally, uncertainty was assessed through relative entropy measures—Bhattacharyya, Kullback–Leibler, Hellinger, and Jeffreys—to evaluate the sensitivity of homogenized properties to input variability. These entropy measures quantify the probabilistic distance between core material distributions and their effective counterparts, reinforcing the importance of precise modeling in the design and optimization of cellular structures.

## 1. Introduction

Cellular materials are widely observed in natural [1,2,3,4] and engineered structures [5,6,7] as a hexagonal structure that exhibits various mechanical properties, including high strength due to their light weight, high energy absorption, and thermal insulation due to their structural configuration or integrity. Various studies discussed cellular material as a hexagonal structure in the form of honeycombs, lattice structures, and foam, which play a significant role in multiple conditions [5,6,7,8,9,10,11,12,13,14,15].

Several studies have investigated the mechanical properties of hexagonal cellular structures. Yu et al. (2023) [16] identified topological defects, specifically five to seven defects, in hexagonal honeycombs through observations of heterogeneous structures in nature, such as graphene, insect eyes, and beehives; Swellam et al. (1997) [10] examined the mechanical behavior of foam cells within a series of hexagonal configurations; Habib et al. (2018) [8] analyzed the compressive behavior and strain energy of nine types of honeycomb structures, including the hexagonal configuration; Liu et al. (2021) [17] numerically investigated the in-plane crushing behavior of hexagonal honeycombs, focusing on the influence of different Poisson’s ratio values; and Carlsson et al. (2022) [18] provided a numerical solution for the elastic–plastic response of hexagonal unit cells subjected to uniaxial tensile and compressive loading. In light of these literature studies, the present work conducts analytical and numerical homogenization to evaluate the Young’s modulus and yield strength of the hexagonal unit structure.

Homogenization techniques play a crucial role in the complex geometry and boundary conditions of cellular materials. Various approaches to homogenization, such as analytical and numerical methods, have been used to determine the effective mechanical properties of cellular materials [19,20,21,22,23]. Analytical homogenization based on the assumption of periodic microstructure and idealization of the unit cell, such as square, hexagonal, tetrakaidekahedral, etc. The basic principles of mechanics, such as beam theory [24,25,26], strain energy method [27,28,29], Gibson–Ashby Model [30,31], asymptotic [21,32,33], and closed-form expressions for effective moduli [17,34], were used to derive a mathematical equation as a function of geometric parameters and base material properties. Most analytical homogenization of the effective mechanical properties of cellular materials yields linear results due to the following assumptions: uniform material distribution, linear elasticity, and a regular periodic structure. While numerical homogenization often employs the finite element method (FEM), providing a more general and accurate framework, this approach considers the representative volume element (RVE) and subjects it to specified boundary conditions and various meshing analyses, including quad, quad-dominated, and triangular meshes [23,35,36,37,38].

In cellular material, the geometric parameters of the unit cell, such as length [24,39,40,41], nodal connection [41,42,43], angle between nodes [4,25,44], thickness of the cell wall [31,39], and its width, play an essential role in the mechanical properties of the entire cellular solid. Additionally, the mechanical properties vary with the base material, which can be homogeneous or non-homogeneous [8,25]. In both cases, geometric topology and base material properties are crucial for optimizing and designing the cellular material; in this way, analytical and numerical homogenization are essential for predicting the effective mechanical properties as factors vary.

Cellular materials are widely studied for their excellent energy absorption in vehicle crashworthiness applications [45], but challenges remain in optimizing geometry and material combinations for consistent crash performance. Fatigue failure of platform screen doors under subway aerodynamic loads further highlights the need for structural systems that can dissipate energy and resist cyclic stresses [46]. Recently, hybrid friction energy-absorbing structures (HFEAS), comprising a bearing bush, anti-creeper device, pretension bolt, friction plate, and frictional metal/CFRP tubes, have emerged as a promising solution by combining frictional dissipation with structural compliance [47]. However, the integration of frictional mechanisms with cellular architectures remains unexplored, leaving a clear gap in developing multifunctional systems that combine crashworthiness, fatigue resistance, and reliable energy absorption for enhanced transportation safety. Within this context, 2D hexagonal cellular solids are particularly attractive because of their simple geometry, tunable mechanical properties, and resemblance to natural honeycomb structures. However, in practical applications, uncertainties inevitably arise from variability in material properties, manufacturing imperfections, and geometric irregularities, all of which can significantly influence their performance and reliability [48]. Accurate uncertainty quantification of the mechanical properties of hexagonal cellular solids is therefore crucial for robust design and safe deployment in engineering systems. Despite extensive studies on their deterministic mechanical behavior, limited research has systematically addressed stochastic aspects, especially through combined analytical and finite element method (FEM) approaches [49,50,51]. This gap motivates the present work, which utilizes Monte Carlo simulation, stochastic perturbation technique, and semi-analytical methods to perform an uncertainty analysis of both analytical and numerical models of cellular solids.

This study presents a systematic framework for the uncertainty quantification of the mechanical properties of 2D hexagonal cellular solids using a combined analytical and finite element method (FEM) approach. Variability in parameters such as cell wall thickness, elastic modulus, and geometric irregularities is modeled and propagated to quantify the stochastic response of the effective elastic and strength properties. Analytical homogenization methods are employed for computational efficiency, while FEM simulations provide high-fidelity verification and benchmarking of the probabilistic predictions. The originality of this work lies in bridging deterministic homogenization with stochastic modeling, highlighting the accuracy and limitations of analytical theories under uncertain inputs.

## 2. Analytical Model

**Figure 1:**
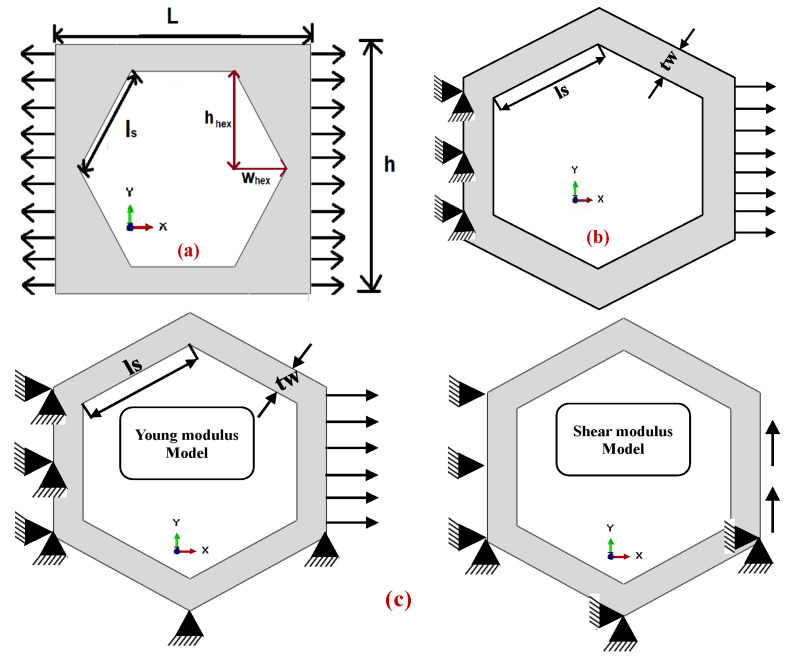
Boundary conditions for (**a**) elastic test, (**b**) plastic test, and (**c**) Poisson’s ratio.

The analytical approach for calculating material parameters for the 2D hexagonal unit cell is based on the elastic bar model. It is divided into three independent testing phases, each devoted to determining the elastic modulus, yield strength, and deriving the effective Poisson’s ratio. The mathematical model for each phase is based on the assumption that the elastic phase of the skeleton characterizes the geometric ability to return to its original shape after deformation, the plastic phase describes the yield strength, and that Poisson’s ratio quantifies the material’s lateral strain relative to axial strain under applied stress. The von Mises metal plasticity model is applicable in this study to model nonlinear deformations of such a solid. The geometric model of the skeletal material under consideration is shown in Figure 1. Let us adopt the following notation: $E_{eff}$ is the analytical Young’s modulus, $E_{m}$ is the Young’s modulus of the base material, $\sigma_{y}$ is the analytical yield strength, $\sigma_{ym}$ is the yield strength of the base material, $\sigma_{T}$ is the tensile yield strength, $\sigma_{C}$ is the compressive yield strength, $\upsilon_{Hex}$ is the analytical Poisson’s ratio, $t_{w}$ is the thickness of the cell wall, $l_{s}$ is the segmental length of cell, L is the external length of unit rectangle, θ is the angle b/w applied force the segment or beam of the hexagon, $h_{hex}$ is the hexagon internal height in vertical direction, $w_{hex}$ is the width of hexagon on horizontal direction, H is the height of unit rectangle, F is the applied unit force, $F_{x}$ is the horizontal component of applied force, $F_{y}$ is the vertical component of applied force, $\varepsilon_{y}$ is the longitudinal strain and $\varepsilon_{x}$ is the transverse strain.

The homogenized Young’s modulus of the two-dimensional hexagonal lattice structure was determined using the analytical geometric homogenization method (also known as the micromechanical analytical approach) [21,52]. In this approach, the hexagonal cell was considered as a periodic representative volume element (RVE), and the overall stiffness was derived based on the geometric configuration and load transfer mechanisms of the cell struts. Let us begin with the mathematical expression describing the horizontal and vertical elements of the cell with applied deformation in the segmental length of hexagon ($l_{s}$) as

(1) $$h_{hex}=l_{s}\sin\theta ,\;\;\;w_{hex}=l_{s}\cos\theta ,\;\;\;A_{eff}=6h_{hex}w_{hex}=6l_{s}^{2}\sin\theta \cos\theta$$

Therefore, the effective Young’s modulus in terms of the homogenized Young’s modulus ($E_{hom}$) and effective area ($A_{eff}$) is

(2) $$E_{eff}=E_{hom}\left[A_{eff}\right].$$

Thus, the final analytical equation of the effective Young’s modulus proceeds using the following equivalence:

(3) $$\left\{\begin{array}{l} A_{eff}=\frac{A_{total}-A_{hole}}{A_{total}}=\left(1-\frac{A_{hole}}{Lh}\right)=\left(1-\frac{2.3l_{s}^{2}\sin\theta \cos\theta }{Lh}\right)\\ \Rightarrow E_{eff}=E_{hom}\left[1-\frac{6l_{s}^{2}\sin\theta \cos\theta }{L\;h}\right] \end{array}\right..$$

Hence, the analytical geometric method provides a simplified yet accurate estimation of the equivalent Young’s modulus for cellular structures.

The effective yield strength [7] of the 2D hexagonal lattice was also derived using the analytical geometric method combined with the yield criterion approach. The cell walls were modeled as slender beam elements subjected to combined axial and shear stresses. By applying the principle of static equivalence and the von Mises yield criterion, the effective analytical yield strength of the lattice can be expressed as

(4) $$\sigma_{ym}=\sigma_{T}+\sigma_{c}\to \sigma_{ym}=\frac{F}{t_{w}^{2}}+\frac{6F}{t_{w}^{2}}.$$

Thus, the maximum force at which the cellular structure exhibits irreversible yielding ($\sigma_{y}$) is can be expressed as

(5) $$\sigma_{y}=\frac{F}{A}\Rightarrow F=\sigma_{y}A\Rightarrow F=\frac{3\sqrt{3}}{2}\sigma_{y}l_{s}^{2}.$$

Substituting the value of force (F) from (5) into (4), one can develop the final equation for yield strength as [7]

(6) $$\left\{\begin{array}{l} \sigma_{ym}=\frac{\sigma_{y}\left[\frac{3\sqrt{3}}{2}l_{s}^{2}\right]}{t_{w}^{2}}+\frac{6\sigma_{y}\left[\frac{3\sqrt{3}}{2}l_{s}^{2}\right]}{t_{w}^{2}}\\ \sigma_{y}=\frac{4\sqrt{3}\sigma_{ym}t_{w}^{2}}{21\left[l_{s}^{2}\right]} \end{array}\right..$$

The homogenized Poisson’s ratio for the hexagonal lattice structure was also obtained through the analytical geometric method by analyzing the deformation kinematics of the hexagonal cell. Under uniaxial loading, the lateral deformation was derived from the rotation and bending of the inclined cell walls. The axial deformation $\Delta_{x}$ with applied force F, bending moment on the M with segmental length $l_{s}$, and end rotation ϕ is expressed as

(7) $$\left\{\begin{array}{l} \Delta_{x}=\varepsilon_{x}l_{s}\\ F=EA\frac{\Delta_{x}}{l_{s}}=Et_{w}\varepsilon_{x},A=1*t_{w}=t_{w}\left(Unit\;Depth\right),I=\frac{1}{12}t_{w}^{3}\\ M=F\frac{l_{s}}{2}\cos30^{\circ }=F\frac{\sqrt{3}}{4}l_{s}\\ \phi =\frac{Ml_{s}}{2EL}=\varepsilon_{x}\frac{3\sqrt{3}}{3}\frac{l_{s}^{2}}{t_{w}^{2}} \end{array}\right..$$

The transverse deformation $\Delta_{y}$ and the macroscopic strain with the regular hexagonal height $\frac{3\sqrt{3}}{4}ls$ is expressed as

(8) $$\left\{\begin{array}{l} \Delta_{y}=2\left(\frac{l_{s}}{2}\phi \sin60^{\circ }\right)=\frac{\sqrt{3}}{2}\phi l_{s}\\ \varepsilon_{y}=-\frac{\Delta_{y}}{\frac{3\sqrt{3}}{4}ls}=-\phi \frac{1}{3} \end{array}\right..$$

The simplification of Equation (8) in terms of axial $\varepsilon_{x}$ and transversal $\varepsilon_{y}$ strain is

(9) $$\varepsilon_{y}=-\frac{1}{3}\left(\frac{t_{w}}{l_{s}}\right)\varepsilon_{x}+{\left(\frac{t_{w}}{l_{s}}\right)}^{3}.$$

Since the term ${\left(\frac{t_{w}}{l_{s}}\right)}^{3}\ll 1$, we neglect this term and Equation (9) is reframed as

(10) $$\varepsilon_{y}=-\frac{1}{3}\left(\frac{t_{w}}{l_{s}}\right)\varepsilon_{x}.$$

The final equation for Poisson’s ratio is given below. According to Ongaro, F. (2018) [49], the value of this coefficient C for a hexagonal structure is 0.33; therefore, Equation (10) becomes

(11) $$\upsilon_{Hex}=-\frac{\varepsilon_{y}}{\varepsilon_{x}}=\frac{1}{3}\left(\frac{t_{w}}{l_{s}}\right)\approx 0.33\left(\frac{t_{w}}{l_{s}}\right).$$

These closed-form equations for the effective material parameters of the elastic–plastic homogeneous and isotropic equivalent solid enable a relatively easy probabilistic model, where the solid skeleton material and geometrical parameters become random variables and/or fields.

## 3. Numerical Analysis

Consider a static equilibrium of a statistically homogeneous and bounded continuum $\Omega \subset {ℜ}^{2}$. Let us assume further that there are non-empty subsets of the external boundaries of this region Ω, namely ∂Ω and $\partial \Omega_{u}$, where the Dirichlet and von Neumann boundary conditions are defined, correspondingly. They result in a displacement field and stress tensor components that satisfy the linear elasticity elliptic boundary value problem given by Equations (12)–(16). It should be emphasized that the proposed homogenization method does not require any Dirichlet boundary conditions in contrast to the approach presented in (Kamiński, 2005; Kamiński, 2013) [53,54]; they are introduced for a brevity of presentation. Considering the proposed numerical technique, the entire set of homogenization boundary value problems with the same boundary conditions and with additionally modified input variables (Kamiński, 2005) [53] is to be solved, where *n* represents the number of discrete realizations of the mean value. The upper index α indicates the different structural responses associated with these input values. The solution to the particular boundary differential equation systems that describe the static equilibrium near the average value of this parameter is sought:

(12) $$\sigma_{ij}^{\left(\alpha \right)}(x)=C_{ijkl}^{\left(\alpha \right)}\varepsilon_{kl}^{\left(\alpha \right)}(x),$$

(13) $$\varepsilon_{ij}^{\left(\alpha \right)}(x)=\frac{1}{2}\left(\frac{\partial u_{i}^{\left(\alpha \right)}(x)}{\partial x_{j}}+\frac{\partial u_{j}^{\left(\alpha \right)}(x)}{\partial x_{i}}\right),$$

(14) $$\sigma_{ij,j}^{\left(\alpha \right)}(x)=0,$$

(15) $$u_{i}^{\left(\alpha \right)}\left(x\right)={\hat{u}}_{i}\left(x\right);\;x\in \partial \Omega_{u},$$

(16) $$\sigma_{ij}^{\left(\alpha \right)}\left(x\right)n_{j}={\hat{t}}_{i}\left(x\right);\;x\in \partial \Omega_{\sigma }.$$

The elasticity tensor of the core material satisfies the symmetry, boundedness, and ellipticity conditions, and is defined as

(17) $$C_{ijkl}\left(x\right)=e\left(x\right)\left\{\delta_{ij}\delta_{kl}\frac{\nu \left(x\right)}{\left(1+\nu \left(x\right)\right)\left(1-2\nu \left(x\right)\right)}+(\delta_{ik}\delta_{jl}+\delta_{il}\delta_{jk})\frac{1}{2\left(1+\nu \left(x\right)\right)}\right\},$$

where $E\left(x\right)$ and $\nu \left(x\right)$ denote the Young’s modulus and Poisson’s ratio, respectively, with i,j,k,l=1,2. The following series variational formulation is introduced and discretized to obtain an appropriate numerical solution with the finite element method:

(18) $$\int_{\Omega }C_{ijkl}^{\left(\alpha \right)}u_{i,j}^{\left(\alpha \right)}\delta u_{k,l}^{\left(\alpha \right)}d\Omega =\int_{\partial \Omega_{\sigma }}{\hat{t}}_{i}\delta u_{i}^{\left(\alpha \right)}d\left(\partial \Omega \right),$$

The left-hand side of Equation (18) corresponds to the elastic behavior of the structure, and the right-hand side is equivalent to the applied stress boundary conditions. It should be noted that the indexing with α, in addition to the Response Function Method (RFM), should be incorporated into the computational domain *Ω* for modeling shape sensitivity; the corresponding extension and additional conditions may reflect fluctuations in this external boundary structure. The determination of the effective material tensor requires the introduction of the strain energy for a heterogeneous medium, i.e.,

(19) $$U^{\left(\alpha \right)}\equiv U\left(h^{\left(\alpha \right)}\right)=\frac{1}{2}\int_{\Omega }C_{ijkl}^{\left(\alpha \right)}\varepsilon_{ij}^{\left(\alpha \right)}\varepsilon_{kl}^{\left(\alpha \right)}d\Omega .$$

The homogenized medium accumulates the same amount of energy as a medium with a series of effective elastic characteristics $C_{ijkl}^{\left(eff\right)\left(\alpha \right)}$, so that we can compare this with the strain energy stored in the original medium:

(20) $$U^{\left(\alpha \right)}=\frac{1}{2}\int_{\Omega }C_{ijkl}^{\left(\alpha \right)}\varepsilon_{ij}^{\left(\alpha \right)}\varepsilon_{kl}^{\left(\alpha \right)}d\Omega =U^{\mathrm{hom}\left(\alpha \right)}=\frac{1}{2}\int_{\Omega }C_{ijkl}^{\left(eff\right)\left(\alpha \right)}\varepsilon_{ij}^{x\left(\alpha \right)}\varepsilon_{kl}^{x\left(\alpha \right)}d\Omega ,$$

where $\varepsilon_{ij}^{x\left(\alpha \right)}$ are the macro strains in the equivalent macro-homogeneous body. The following boundary conditions are applicable in this case:

(21) $$\begin{array}{l} \varepsilon_{ij}^{x_{1}}:\;\;u_{1}\left(l_{1},x_{2},x_{3}\right)=\Delta_{1},\;\;\;\;\;\;u_{2}\left(x_{1},l_{2},x_{3}\right)=0,\;\\ \;\;\;\;\;\;u_{1}\left(-l_{1},x_{2},x_{3}\right)=-\Delta_{1},\;u_{2}\left(x_{1},-l_{2},x_{3}\right)=0, \end{array}$$

and, independently, along the *x*_2_ axis:

(22) $$\begin{array}{l} \varepsilon_{ij}^{x_{2}}:\;\;u_{1}\left(l_{1},x_{2},x_{3}\right)=0,\;\;\;\;\;\;u_{2}\left(x_{1},l_{2},x_{3}\right)=\Delta_{2},\;\;\\ \;\;\;\;\;\;u_{1}\left(-l_{1},x_{2},x_{3}\right)=0,\;\;\;u_{2}\left(x_{1},-l_{2},x_{3}\right)=-\Delta_{2}. \end{array}$$

The macro strains within the RVE can be determined as

(23) $$\varepsilon_{ij}^{x_{1}}=\frac{\Delta_{1}}{l_{1}},\;\;\;\varepsilon_{ij}^{x_{2}}=\frac{\Delta_{2}}{l_{2}}$$

and reduced to *x*_1_ and *x*_2_ by only, while assuming additionally uniform size *l* in both directions.

Then, the following system of linear algebraic equations is solved symbolically in the MAPLE system to compute the effective characteristics:

(24) $$\left\{\begin{array}{c} \frac{1}{2}C_{1111}^{\left(eff\right)\left(\alpha \right)}{\left(\varepsilon_{11}^{x_{1}}\right)}^{2}=U_{x_{1}}\equiv U_{1}\\ \frac{1}{2}C_{2222}^{\left(eff\right)\left(\alpha \right)}{\left(\varepsilon_{22}^{x_{2}}\right)}^{2}=U_{x_{2}}\equiv U_{2}\\ \frac{1}{2}\left\{C_{1111}^{\left(eff\right)\left(\alpha \right)}{\left(\varepsilon_{11}^{x_{1}}\right)}^{2}+2C_{1122}^{\left(eff\right)\left(\alpha \right)}\varepsilon_{11}^{x_{1}}\varepsilon_{22}^{x_{2}}+C_{2222}^{\left(eff\right)\left(\alpha \right)}{\left(\varepsilon_{22}^{x_{2}}\right)}^{2}\right\}=U_{x_{1}\cup x_{2}}\equiv U_{12} \end{array}\right.,$$

where the strain energy is equal to

(25) $$U_{x_{1}\cup x_{2}}=U_{x_{1}}+U_{x_{2}}+\frac{1}{2}\int_{\Omega }\sigma_{ij}^{x_{1}}\varepsilon_{ij}^{x_{2}}d\Omega +\frac{1}{2}\int_{\Omega }\sigma_{ij}^{x_{2}}\varepsilon_{ij}^{x_{1}}d\Omega$$

in the case of biaxial tension of the RVE. The computational implementation in the framework of the finite element method is relatively straightforward and follows directly from traditional approaches (see, e.g., Kamiński, 2013) [54,55,56,57,58,59].

We provide in turn a mathematical basis for the Least-Squares Method (LSM) adjacent to the fourth-order tensor in both the non-weighted (NLSM) and weighted (WLSM) versions of the homogenization method (Kamiński, 2013) [54]. We use a polynomial approximation of the *s*th order (indexed by *β* here) through n numerical tests of the homogenization problem solved near the mean value of the given design parameter h. As a result, we obtain *n* different pairs $\left(h_{\alpha },C_{ijkl}^{\left(eff\right)\left(\alpha \right)}\right)$α=1,…,n. We seek the following polynomial representation of the effective tensor versus the given design parameter:

(26) $$C_{ijkl}^{\left(eff\right)}≅D_{ijkl}^{\left(\beta \right)}h^{\beta }=f\left(D_{ijkl},h\right)\;\;\;\;\beta =1,\ldots ,s;\;s<n;\;\;\;i,j,k,l=1,2.$$

We introduce for this purpose the residuals at each trial point and each component of the homogenized tensor, i.e.:

(27) $$r_{ijkl\left(\alpha \right)}=C_{ijkl}^{\left(eff\right)\left(\alpha \right)}-f\left(D_{ijkl},h_{\alpha }\right)\;\;\;\;\alpha =1,\ldots ,n;\;\;\;i,j,k,l=1,2.$$

The coefficients $D_{ijkl}$ are found by minimizing the weighted residual function:

(28) $$S_{ijkl}=\sum_{\alpha =1}^{n}w_{\alpha \alpha }r_{ijkl\left(\alpha \right)}^{2}\;\;\;\;\alpha =1,\ldots ,n;\;\;\;i,j,k,l=1,2.$$

Such a minimization can be performed as follows (Björck, 1996) [57,60]:

(29) $$\frac{\partial S_{ijkl}}{\partial D_{ijkl}^{\left(\beta \right)}}=-2\sum_{\alpha =1}^{n}w_{\alpha \alpha }r_{ijkl\left(\alpha \right)}\frac{\partial f\left(D_{ijkl},h_{\alpha }\right)}{\partial D_{ijkl}^{\left(\beta \right)}}\;\;\;\;\beta =1,\ldots ,s;\;\;\;\;i,j,k,l=1,2.$$

Adopting the following notation for the Jacobian:

(30) $$J^{ijkl}=J_{\alpha \beta }^{ijkl}=\frac{\partial f\left(D_{ijkl},h_{\alpha }\right)}{\partial D_{ijkl}^{\left(\beta \right)}}\;\;\;\;\alpha =1,\ldots ,n;\;\;\;\beta =1,\ldots ,s;\;\;\;\;i,j,k,l=1,2.$$

one can get

(31) $$\sum_{\alpha =1}^{n}\sum_{\beta =1}^{s}J_{\alpha \beta }^{ijkl}w_{\alpha \alpha }J_{\alpha \beta }^{ijkl}D_{ijkl}^{\left(\beta \right)}=\sum_{\alpha =1}^{n}J_{\alpha \beta }^{ijkl}w_{\alpha \alpha }C_{ijkl}^{(eff)\left(\alpha \right)},\;\;\;\;\;\alpha =1,\ldots ,n;\;\beta =1,\ldots ,s;\;i,j,k,l=1,2.$$

Thus, we obtain the following matrix of normal equations:

(32) $$\left({\left(J^{ijkl}\right)}^{T}w\;J^{ijkl}\right)\;D^{ijkl}={\left(J^{ijkl}\right)}^{T}w\;C_{ijkl}^{\left(eff\right)},\;\;\;\;i,j,k,l=1,2.$$

This system of equations is solved symbolically in the system MAPLE for three components of the effective elasticity tensor, namely $C_{1111}^{\left(eff\right)}$, $C_{1122}^{\left(eff\right)}$, and $C_{1212}^{\left(eff\right)}$, while determining the orthotropic effective medium for the given hexagonal material. The situations simplify in the case of an isotropic homogenized medium, which is investigated below, and where direct approximation of Young’s modulus and Poisson’s ratio is completed.

## 4. Computer Simulation

The analytical and numerical homogenization approaches for a hexagonal unit cell are employed here to determine the effective mechanical parameters, including Young’s modulus, yield strength, and Poisson’s ratio. This analysis was performed by introducing slight variations, both increases and decreases, in key geometric parameters such as segment length, cell wall thickness, and material properties of the unit cell. Analytical approaches have been implemented directly in the symbolic algebra package MAPLE 2025.1, whereas the FEM analysis was delivered in the system ABAQUS/CAE 6.14-5.

**Figure 2:**
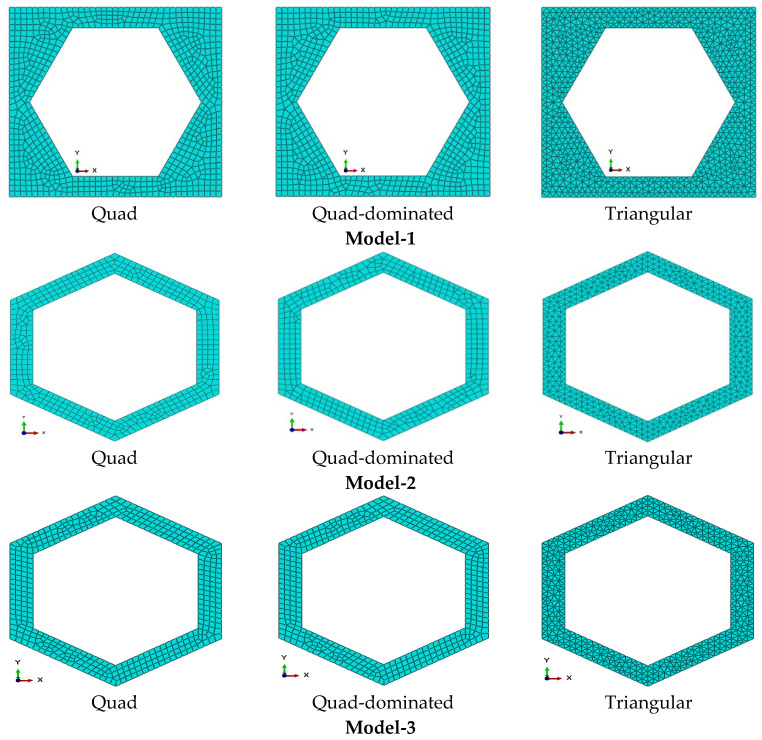
Meshing schemes in various FEM models of hexagonal solid.

Some specific geometric dimensions of the hexagonal unit cell used in the numerical simulations were employed to calculate the corresponding values of Young’s modulus, yield strength, and Poisson’s ratio. The segmental length ranged from 4.41 mm to 4.71 mm, and the material’s Young’s modulus ranged from 66.5 GPa to 71.5 GPa. A similar procedure was followed for determining the yield strength and Poisson’s ratio, incorporating segment length variations of 0.3 mm (4.41 to 4.71 mm) and cell wall thickness variations of 0.01 mm (1.04 to 1.14 mm). Three types of meshing were employed in ABAQUS, as shown in Figure 2, namely quadrilateral (Quad, quad-dominated) and triangular meshes, with the principal aim of collecting reliable numerical results that closely correspond to the analytical predictions, thereby validating the accuracy and robustness of the homogenization methodology.

**Figure 3:**
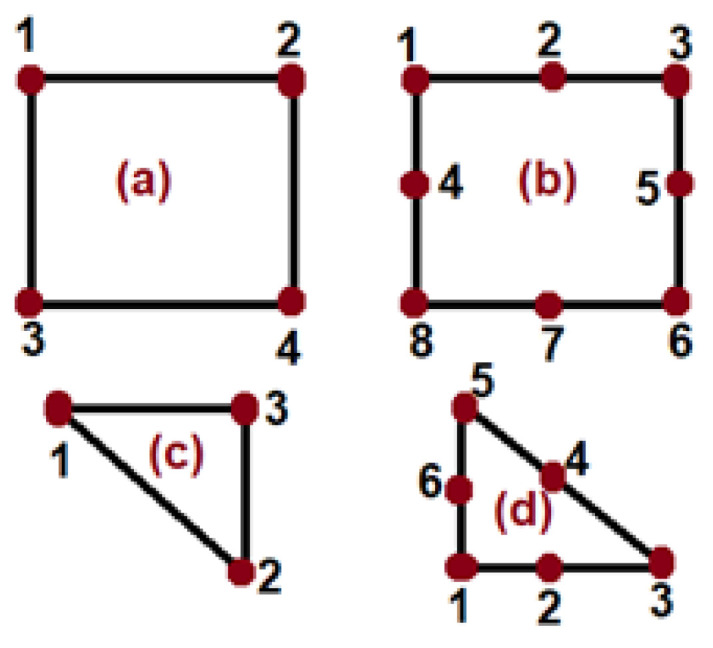
(**a**) Quadrilateral 4 node element, (**b**) quadrilateral 8 node element, (**c**) triangular 3 node element, and (**d**) triangular 6 node element.

In numerical homogenization, a uniform mesh element size of 0.3 is used across all models. These models are categorized based on mesh element geometry and the number of nodes per element. Each case of the model has distinct mesh geometries: quadrilateral (Quad), quad-dominated, and triangular, with 4, 4, and 3 nodes per element, and for more precise numerical results, each finite element is further divided into 8, 8, and 6 nodes per element in the quadrilateral (Quad), Quad-dominated, and triangular cases, respectively. The meshing configurations and number of nodes for all three models are shown in Figure 2 and Figure 3, respectively, and also the total number of mesh elements and nodes of each model as shown in Appendix A.

In all three-phase testing of the numerical model, aluminum material was assigned with Young’s modulus of 69 GPa and a yield strength of 69 MPa in the case of the plastic test. In the numerical measurement of an elastic model, Young’s modulus is determined using the following expression:

(33) $$E_{eff}^{FEM}=\left[\frac{\sigma_{left-x-axis}+\sigma_{right-x-axis}}{\varepsilon_{left-x-axis}+\varepsilon_{right-x-axis}}\right]$$

where $E_{eff}^{FEM}$ is numerical Young’s modulus, $\sigma_{left-x-axis},\sigma_{right-x-axis}$ is stress on the right and left side, and $\varepsilon_{left-x-axis},\varepsilon_{right-x-axis}$ is strain on the right and left side. Finally, Poisson’s ratio is quantified numerically using the following expression:

(34) $$G_{eff}=\frac{E_{eff}}{2(1+\upsilon_{eff})}\Rightarrow \upsilon_{eff}=\frac{E_{eff}}{2G_{eff}}-1$$

**Figure 4:**
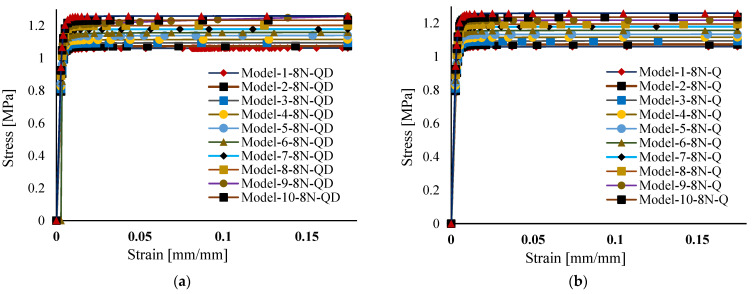
Stress–strain curve of (**a**) thickness $\left(t_{w}\right)$ series of quadrilateral 8 node elements and (**b**) thickness $\left(t_{w}\right)$ series of quadrilateral 8 node elements.

**Figure 5:**
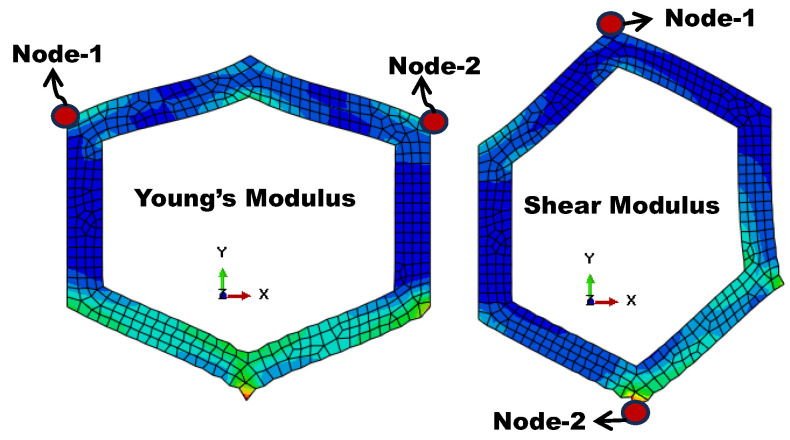
Deformed shape in longitudinal and shear deformation models.

Hence,

(35) $$E_{eff}=\frac{\sigma_{axial}}{\varepsilon_{axial}},G_{eff}=\frac{\sigma_{Shear}}{\varepsilon_{Shear}}$$

where $\sigma_{axial}$ and $\sigma_{shear}$ represent axial and shear stress, respectively, and $\epsilon_{axial}$ and $\epsilon_{shear}$ represent axial and shear strain. Numerically, the yield strength is obtained from the stress–strain curve, where the point at which yielding begins is taken as the yield strength of the model. The stress–strain curves for the various models, generated using different mesh types and varying numbers of nodes, are shown in Figure 4. The axial and shear strains are determined by the distance between nodes 1 and 2 before applying the load, divided by the distance between nodes 1 and 2 after the load (deformed shape), as shown in Figure 5.

**Figure 6:**
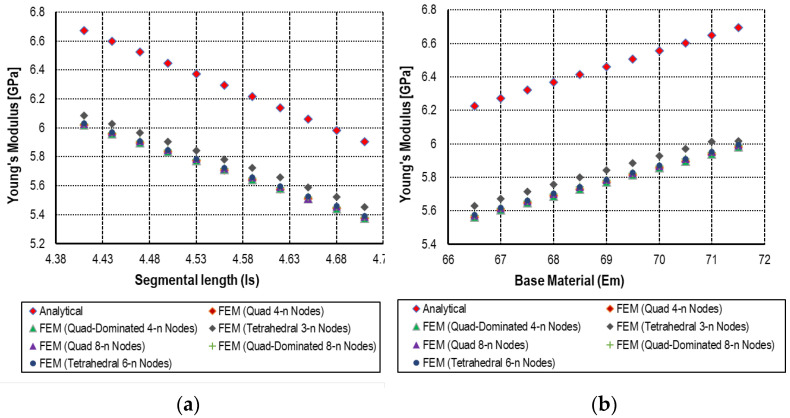
Analytical and numerical result of Young’s modulus with a series of (**a**) segmental $\left(l_{s}\right)$ length and (**b**) material properties $\left(E_{m}\right)$ of hexagonal unit cell.

**Figure 7:**
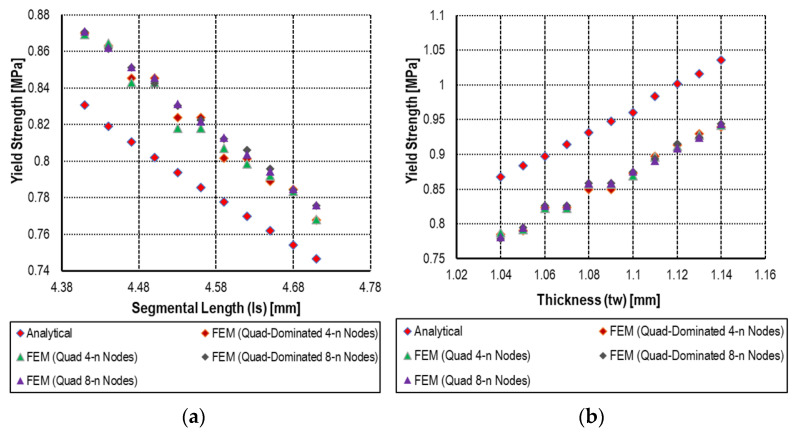
Analytical and numerical result of yield strength with a series of (**a**) segmental length $\left(l_{s}\right)$ and (**b**) thickness of cell wall $\left(t_{w}\right)$ of hexagonal unit cell.

**Figure 8:**
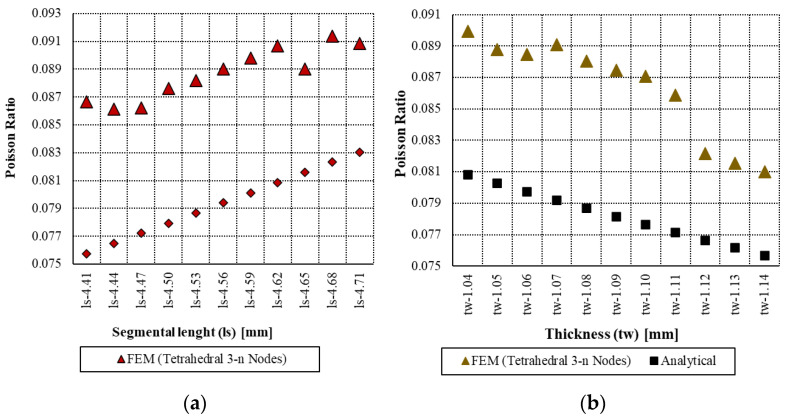
Analytical and numerical results of Poisson with a series of (**a**) segmental length $\left(l_{s}\right)$ and (**b**) thickness of cell wall $\left(t_{w}\right)$ of hexagonal unit cell.

The most important notice related to the effective mechanical properties (Young’s modulus, yield strength, and Poisson’s ratio) of the cellular solid exhibit distinct relationships with geometric parameters and material properties when subjected to small incremental variations. Analytically, as described by the mathematical formulation of Young’s modulus in Equation (3), a clear inverse relationship exists between Young’s modulus and the segmental length $\left(l_{s}\right)$, while a direct relationship was observed with the material’s intrinsic Young’s modulus. Accordingly, as the segmental length increases, the effective Young’s modulus decreases, which is consistent with the trends observed in the numerical simulations as shown in Figure 6. Similarly, an increase in the material’s Young’s modulus $\left(E_{m}\right)$ leads to a corresponding increase in the effective Young’s modulus. Quite similarly, the relationship of yield strength and Poisson’s ratio with incremental variations in segmental length $\left(l_{s}\right)$ and cell wall thickness $\left(t_{w}\right)$ reveals distinct trends in the mechanical response of the hexagonal cellular structure as shown in Figure 7 and Figure 8, respectively. Analytically, these relationships are defined by Equations (6) and (11) for yield strength and Poisson’s ratio, respectively. As shown in Figure 6, Figure 7 and Figure 8, both properties exhibit an inverse correlation with segmental length, indicating that as $l_{s}$ increases, the corresponding values of yield strength and Poisson’s ratio decrease. Conversely, a direct relationship is observed with respect to the cell wall thickness $\left(t_{w}\right)$, where increases in $\left(t_{w}\right)$ lead to an increase in both yield strength and Poisson’s ratio.

In our numerical simulations, we primarily selected quadrilateral (Quad) and Quad-dominated meshes for evaluating Young’s modulus and yield strength, since these meshes produced results closest to the analytical solutions. This is because Quad elements are generally more accurate for representing axial and shear responses under uniform loading conditions, leading to better convergence in stiffness-related properties such as Young’s modulus and yield strength. However, in the case of Poisson’s ratio, triangular meshes provided results closer to the analytical values. This can be attributed to the ability of triangular elements to better capture localized deformations and lateral strain distributions, which play a critical role in accurately estimating Poisson’s ratio.

The analytical results for Young’s modulus demonstrate a decreasing trend from 6.6725 GPa to 5.9036 GPa as the incremental variation in the segmental length $\left(l_{s}\right)$ from 4.41 mm to 4.71 mm. Accompanying numerical results show a slightly less pronounced decline, from 6.019 GPa to 5.392 GPa. This discrepancy may be attributed to the simplifications inherent in the analytical model, which assumes idealized boundary conditions, whereas the numerical model incorporates more realistic constraints and deformation behavior. Furthermore, when varying the base material’s Young’s modulus $\left(E_{m}\right)$ from 66.5 GPa to 71.5 GPa, the analytically determined effective Young’s modulus increases from 6.2275 GPa to 6.6957 GPa, while the numerical values range from 5.563 GPa to 5.996 GPa. This slight deviation is likely due to mesh sensitivity and stress concentration effects captured in the finite element analysis, which are not accounted for in the analytical expressions.

Although with an incremental variation in segmental length $\left(l_{s}\right)$ from 4.41 mm to 4.71 mm, the analytical results showed a reduction in yield strength from 0.831 MPa to 0.747 MPa, and analogously a decrease in Poisson’s ratio from 0.0808 to 0.0756. Correspondingly, the numerical results show a decline in yield strength from 0.8701 MPa to 0.776 MPa and in Poisson’s ratio from 0.0899 to 0.0810. In the case of incremental variation in cell wall thickness $\left(t_{w}\right)$ from 1.08 mm to 1.14 mm, the analytical yield strength increases from 0.867 MPa to 1.036 MPa, while Poisson’s ratio slightly decreases from 0.0757 to 0.830. And numerically, the yield strength rises from 0.785 MPa to 0.944 MPa, accompanied by an increase in Poisson’s ratio from 0.0866 to 0.0908.

The elastic response was examined through a parametric sweep involving variations in segmental length and material modulus, revealing percentage differences between analytical and numerical results ranging from −8.28% to 10.87% and from −10.58% to 11.95%, respectively. Similarly, yield strength was evaluated with respect to variations in segmental length and wall thickness, showing discrepancies between −2.86% to −5.53% and 7.76% to 10.57%, respectively. For Poisson’s ratio, variations in the same parameters led to differences ranging from −7.05% to −12.48% and −9.11% to −12.64%, respectively.

The discrepancies between analytical and numerical results of the effective mechanical parameters, such as Young’s modulus, yield strength, and Poisson’s ratio of hexagonal cellular material, can be attributed to several factors. In the case of an analytical model, we assumed a uniform material distribution, linear elastic behavior, and idealized geometry that may not fully capture the stress distribution and deformation percentage in the actual structure. While in numerical simulation (FEM), consider geometric non-linearities, different stress distributions, and more accurate localized plastic deformation, which leads to a realistic representation of effective mechanical behavior. Additionally, mesh density, type of mesh element, and boundary conditions in the numerical simulation (FEM) can influence the output result.

## 5. Development of Polynomial Model

Based on variations in material properties, segmental length, and cell wall thickness in both analytical and numerical models, we obtained multiple sets of results. From these results, six-order polynomial models for all effective parameters (Young’s modulus, yield strength, and Poisson’s ratio) were generated using trend analysis in MS Excel. The resulting sixth-order polynomial expressions are presented below, and the corresponding regression statistics are summarized in Table 1.

The following polynomials have been determined:

Polynomial for Young’s Modulus in the case of variation with material property $\left(E_{m}\right)$:

(36) $$\left\{\begin{array}{l} y_{Analytical}\;=\;5E-20x^{6}-1E-09x^{5}+2E-07x^{4}-1E-05x^{3}+0.0003x^{2}+0.0044x+6.2184\\ y_{Numerical}\;=-2E-11x^{6}+3E-09x^{5}-1E-07x^{4}+1E-06x^{3}+6E-05x^{2}+0.0057x+\;5.5631 \end{array}\right.$$

Polynomial for Young’s Modulus in case of variation with segmental length $\left(l_{s}\right)$:

(37) $$\left\{\begin{array}{l} y_{Analytical}\;=\;5E-12x^{6}+1E-09x^{5}-3E-07x^{4}+2E-05x^{3}-0.0005x^{2}-0.0072x\;+6.6871\\ y_{Numerical}\;=-3E-11x^{6}+9E-09x^{5}-9E-07x^{4}+4E-05x^{3}-0.0009x^{2}-0.0007x+6.041 \end{array}\right.$$

Polynomial for Yield strength in case of variation with segmental length $\left(l_{s}\right)$:

(38) $$\left\{\begin{array}{l} y_{Analytical}\;=9E-11x^{6}-1E-08x^{5}+7E-07x^{4}-2E-05x^{3}+0.0003x^{2}-0.004x+0.8346\\ y_{Numerical}\;=3E-10x^{6}-3E-08x^{5}+1E-06x^{4}-2E-05x^{3}+0.0001x^{2}-0.002x+0.874 \end{array}\right.$$

Polynomial for Yield strength in case of variation with thickness of cell wall $\left(t_{w}\right)$:

(39) $$\left\{\begin{array}{l} y_{Analytical}\;=2E-10x^{6}-3E-08x^{5}+1E-06x^{4}-3E-05x^{3}+0.0003x^{2}+0.0026x\;+0.8648\\ y_{Numerical}\;=9E-10x^{6}-1E-07x^{5}+7E-06x^{4}-0.0002x^{3}+0.0025x^{2}-0.009x+0.7919 \end{array}\right.$$

Polynomial for Poisson’s ratio in case of variation with segmental length $\left(l_{s}\right)$:

(40) $$\left\{\begin{array}{l} y_{Analytical}\;=6E-15x^{6}-1E-12x^{5}+\;2E-10x^{4}-3E-08x^{3}+4E-06x^{2}-0.0006x+0.0814\\ y_{Numerical}\;=3E-07x^{6}-9E-06x^{5}+0.0001x^{4}-0.0009x^{3}+0.0034x^{2}-0.0067x+0.094 \end{array}\right.$$

Polynomial for Poisson’s ratio in case of variation with thickness of cell wall $\left(t_{w}\right)$:

(41) $$\left\{\begin{array}{l} y_{Analytical}\;=-8E-17x^{6}+7E-15x^{5}+2E-13x^{4}-8E-12x^{3}+8E-11x^{2}+0.0007x\;+0.075\\ y_{Numerical}\;=-5E-07x^{6}+2E-05x^{5}-0.0002x^{4}+0.0013x^{3}-0.0035x^{2}+0.0037x+0.0854 \end{array}\right.$$

**Figure 9:**
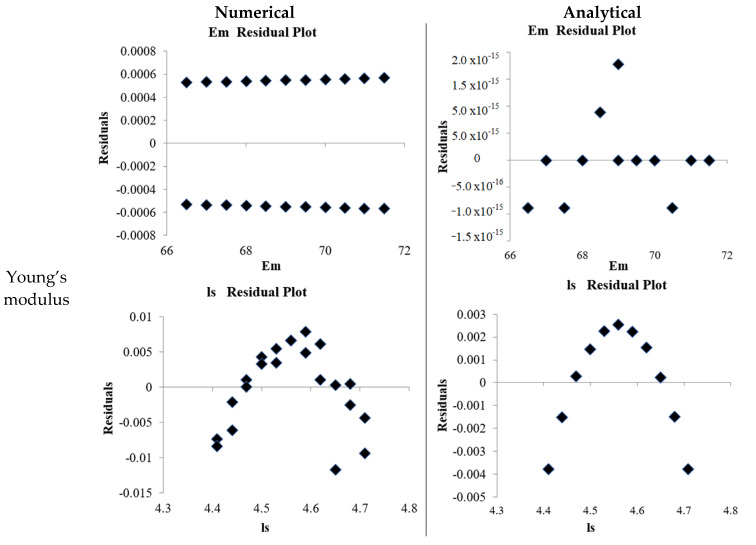

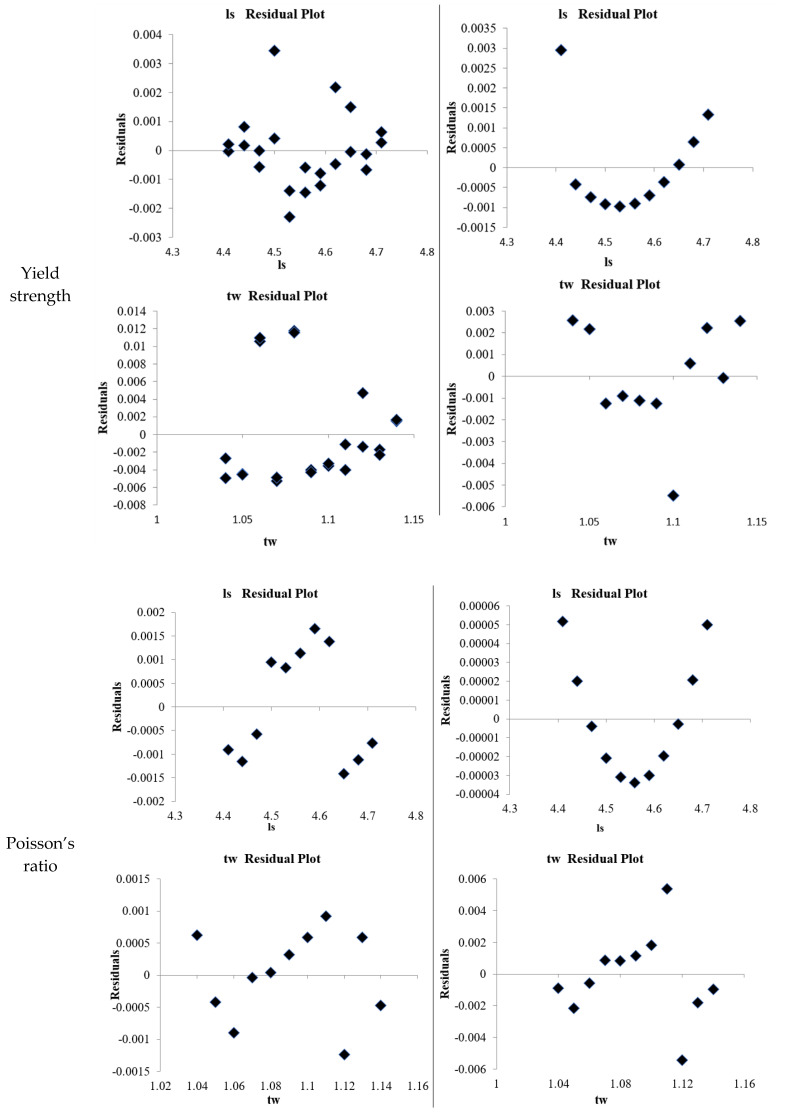
Residual plot of effective mechanical parameters for numerical and analytical results.

The comparative analysis of the regression coefficients (see Appendix B) and residual plots (seen in Figure 9) between the numerical and analytical models reveals that the numerical approach provides a more realistic representation of the effective mechanical parameters. Although the analytical solutions exhibit mathematically perfect precision, as evidenced by their near-zero standard errors and extremely large t-statistics, such results often reflect an idealized system that lacks sufficient accommodation of variability. In contrast, the numerical regressions, while presenting slightly larger error margins, align more closely with physically expected trends and practical observations as shown in Appendix B. For instance, Young’s modulus in the numerical model has the coefficients that are physically interpretable and consistent with realistic material stiffness values. In contrast, the analytical outputs tend to overfit the data space. Similarly, for yield strength, the numerical slope captures the expected sensitivity to geometry while leaving minor but meaningful residuals that reflect the natural scatter of mechanical testing. Poisson’s ratio also shows stronger plausibility in the numerical results, with moderate coefficients that represent realistic lateral contraction effects. At the same time, the analytical regressions tend to exaggerate precision without accounting for system imperfections. Residual graphs reinforce this interpretation: the numerical residuals are randomly scattered around zero without systematic bias, a hallmark of well-fitted but physically constrained models. In contrast, the analytical residuals converge too closely to the regression line, suggesting over-idealization. Therefore, despite the mathematical elegance of the analytical solutions, the numerical regressions provide a more faithful depiction of the material response by accommodating inherent variability and experimental uncertainty, thereby making them more reliable for practical applications.

## 6. Uncertainty Quantification

In this study, the mechanical response of a 2D hexagonal unit cell was modeled using a sixth-order polynomial function representing the nonlinear behavior of an effective mechanical property under varying microstructural conditions (variation in the core material and geometric parameters). Three different computational strategies were employed: the Monte Carlo simulation (MCS), the stochastic perturbation technique (SPT), and a semi-analytical method (SAM). All uncertainty analyses were conducted across a range of randomness parameters, with alpha varying from 0.025 to 0.275 in increments of 0.025. The Monte Carlo simulation, as the reference approach, was implemented to capture the full probabilistic behavior of the unit cell response under uncertainty. A total of 1,000,000 simulation trials were conducted for each value of α, wherein the input random variables (e.g., base material, geometric parameters $\left(t_{w},l_{s}\right)$) were generated from prescribed probability distributions. The sixth-order polynomial model was evaluated numerically for each realization, allowing for the statistical characterization (mean, variance, higher moments) of the output mechanical property. The method did not involve iterative convergence in the classical sense, but instead relied on statistical convergence across the ensemble, with a sufficient sample size ensuring the accurate estimation of probabilistic measures.

The stochastic perturbation technique was implemented to provide a computationally efficient alternative to the full-scale MCS, and mechanical response function was expanded as a Taylor series about the mean values of the input random variables, capturing the effects of stochastic fluctuations up to higher-order terms in this method. The perturbation order was systematically modified from 1 to 10, allowing for an investigation of the sensitivity of response statistics to the level of approximation. For each α increment, the perturbation-based expansion yielded approximations to the first and second statistical moments (mean and variance), with higher-order perturbations offering increased accuracy at the cost of analytical complexity. This method required no iterative numerical solver, as derivatives were evaluated symbolically or via automatic differentiation using MAPLE 2025 symbolic engine. Finally, the semi-analytical method (SAM) was employed as a hybrid approach that integrates analytical differentiation with numerical evaluation. This method involved computing partial derivatives of the sixth-order polynomial with respect to input parameters such as material properties and microstructural variables. These derivatives were then embedded in an incremental-iterative framework to simulate the nonlinear response under varying α. A modified Newton–Raphson algorithm was used for the iterative solution of nonlinear equations, with a convergence tolerance $10^{6}$ based on the norm of residual forces.

Across all three methods, the variation in the parameter α served as a control parameter to examine the influence of microstructural geometry on the global mechanical response. The combination of stochastic and semi-analytical approaches provided both computational efficiency and deep insight into the reliability and robustness of the material system under uncertainty. The comparative analysis of these methods not only validated the consistency of the results but also highlighted the trade-offs between computational cost and solution fidelity. Monte Carlo provided the most accurate statistical results, stochastic perturbation offered efficient moment estimation, and the semi-analytical method enabled deterministic sensitivity analysis with moderate computational effort.

In this study, we also investigate the uncertainty quantification of probabilistic coefficients associated with effective properties. These coefficients are described in terms of statistical parameters (expectation, coefficient of variation, skewness, and kurtosis) and relative entropy measures, specifically, the Bhattacharyya distance, Kullback–Leibler divergence, Jeffreys divergence, and Hellinger distance in a two-dimensional hexagonal cellular unit cell subject to small variations in both base material $\left(E_{m}\right)$ properties and geometric parameters $\left(t_{w},l_{s}\right)$. Uncertainty quantification was achieved through the application of a sixth-order polynomial approximation derived from both analytical and numerical homogenization tests.

Let us consider for this purpose A(x) and N(x) as the analytical and numerical polynomials of 6th order determined by the generalized Taylor expansion, respectively, as follows:

(42) $$A(x)≃\sum_{i=0}^{n}a_{i}x_{i},\;N(x)≃\sum_{i=0}^{n}b_{i}x_{i},\;i=1,2,3\ldots 6$$

where $a_{i}$ and $b_{i}$ are the coefficients of the analytical and numerical polynomials, respectively, and are determined by curve fitting. According to the Bhattacharyya distance $\left(D_{B}[A(x)∥N(x)]\right)$, the polynomial approximation is given as

(43) $$\begin{array}{l} D_{B}[A(x)∥N(x)]=\frac{1}{4}\frac{{\left[E\left(A\left(x\right)\right)-E\left(N\left(x\right)\right)\right]}^{2}}{\sigma^{2}(A(x))+\sigma^{2}(N(x))}+\frac{1}{2}\ln\left(\frac{\sigma^{2}(A(x))+\sigma^{2}(N(x))}{2\sigma (A(x))\sigma (N(x))}\right),\\ D_{B}[A(x)∥N(x)]=\frac{1}{4}\frac{{\left[E\left(a_{i}x^{i}\right)-E\left(b_{i}x^{i}\right)\right]}^{2}}{\sigma^{2}(a_{i}x^{i})+\sigma^{2}(b_{i}x^{i})}+\frac{1}{2}\ln\left(\frac{\sigma^{2}(a_{i}x^{i})+\sigma^{2}(b_{i}x^{i})}{2\sigma (a_{i}x^{i})\sigma (b_{i}x^{i})}\right), \end{array}$$

According to the Kullback–Leibler divergence $\left(D_{KL}[A(x)∥N(x)]\right)$, the polynomial approximation can be proposed in the following way:

(44) $$\begin{array}{l} D_{KL}[A(x)∥N(x)]=\log\left(\frac{\sigma (A(x))E(A(x))}{\sigma (N(x))E(N(x))}\right)+\frac{{\left(\sigma (N(x))E(N(x))\right)}^{2}+{\left(E(A(x))-E(N(x))\right)}^{2}}{2{\left(\sigma (A(x))E(A(x)\right)}^{2}}\\ -\frac{1}{2},\\ D_{KL}[A(x)∥N(x)]=\log\left(\frac{\sigma (a_{i}x^{i})E(a_{i}x^{i})}{\sigma (b_{i}x^{i})E(b_{i}x^{i})}\right)+\frac{{\left(\sigma (b_{i}x^{i})E(b_{i}x^{i})\right)}^{2}+{\left(E(a_{i}x^{i})-E(b_{i}x^{i})\right)}^{2}}{2{\left(\sigma (a_{i}x^{i})E(a_{i}x^{i}\right)}^{2}}\\ -\frac{1}{2}, \end{array}$$

The Hellinger distance $D_{H}[A(x)∥N(x)]$ can be expressed using the following polynomial approximation:

(45) $$\begin{array}{l} D_{H}[A(x)∥N(x)]=1-\sqrt{\frac{\sigma (N(x))E(N(x))\sigma (A(x))E(A(x))}{{\left(\sigma (N(x))E(N(x))\right)}^{2}+{\left(\sigma (A(x))E(A(x))\right)}^{2}}}\\ \exp\left(-\frac{1}{4}\frac{{\left(E(A(x))-E(N(x))\right)}^{2}}{{\left(\sigma (N(x))E(N(x))\right)}^{2}+{\left(\sigma (A(x))E(A(x))\right)}^{2}}\right),\\ D_{H}[A(x)∥N(x)]=1-\sqrt{\frac{\sigma (b_{i}x^{i})E(b_{i}x^{i})\sigma (a_{i}x^{i})E(a_{i}x^{i})}{{\left(\sigma (b_{i}x^{i})E(b_{i}x^{i})\right)}^{2}+{\left(\sigma (a_{i}x^{i})E(a_{i}x^{i})\right)}^{2}}}\\ \exp\left(-\frac{1}{4}\frac{{\left(E(a_{i}x^{i})-E(b_{i}x^{i})\right)}^{2}}{{\left(\sigma (b_{i}x^{i})E(b_{i}x^{i})\right)}^{2}+{\left(\sigma (a_{i}x^{i})E(a_{i}x^{i})\right)}^{2}}\right), \end{array}$$

According to Jeffrey’s divergence $\left(D_{j}[A(x)∥N(x)]\right)$, the polynomial approximation is given as

(46) $$\begin{array}{l} D_{j}[A(x)∥N(x)]=\log\left(\frac{\sigma \left(A(x)\right)E(A(x))}{\sigma \left(N(x)\right)E(N(x))}\right)+\left(\frac{{\left(\sigma \left(N(x)\right)E(N(x))\right)}^{2}+{\left(E(N(x))-E(A(x))\right)}^{2}}{2{\left(\sigma \left(A(x)\right)E(A(x))\right)}^{2}}\right)\\ +\log\left(\frac{\sigma \left(N(x)\right)E(N(x))}{\sigma \left(A(x)\right)E(A(x))}\right)+\left(\frac{{\left(\sigma \left(A(x)\right)E(A(x))\right)}^{2}+{\left(E(N(x))-E(A(x))\right)}^{2}}{2{\left(\sigma \left(N(x)\right)E(N(x))\right)}^{2}}\right)-1\\ D_{j}[A(x)∥N(x)]=\log\left(\frac{\sigma \left(a_{i}x^{i}\right)E(a_{i}x^{i})}{\sigma \left(b_{i}x^{i}\right)E(b_{i}x^{i})}\right)+\left(\frac{{\left(\sigma \left(b_{i}x^{i}\right)E(b_{i}x^{i})\right)}^{2}+{\left(E(b_{i}x^{i})-E(a_{i}x^{i})\right)}^{2}}{2{\left(\sigma \left(a_{i}x^{i}\right)E(a_{i}x^{i})\right)}^{2}}\right)\\ +\log\left(\frac{\sigma \left(b_{i}x^{i}\right)E(b_{i}x^{i})}{\sigma \left(a_{i}x^{i}\right)E(a_{i}x^{i})}\right)+\left(\frac{{\left(\sigma \left(a_{i}x^{i}\right)E(a_{i}x^{i})\right)}^{2}+{\left(E(b_{i}x^{i})-E(a_{i}x^{i})\right)}^{2}}{2{\left(\sigma \left(b_{i}x^{i}\right)E(b_{i}x^{i})\right)}^{2}}\right)-1 \end{array}$$

where E(A(x)) represents the expected value of the analytically derived polynomial, and E(N(x)) represents the expected value of the numerically derived polynomial. Further numerical analysis of these distances has been delivered under the assumption that probability distributions of both properties of the cellular solid are Gaussian.

**Figure 10:**
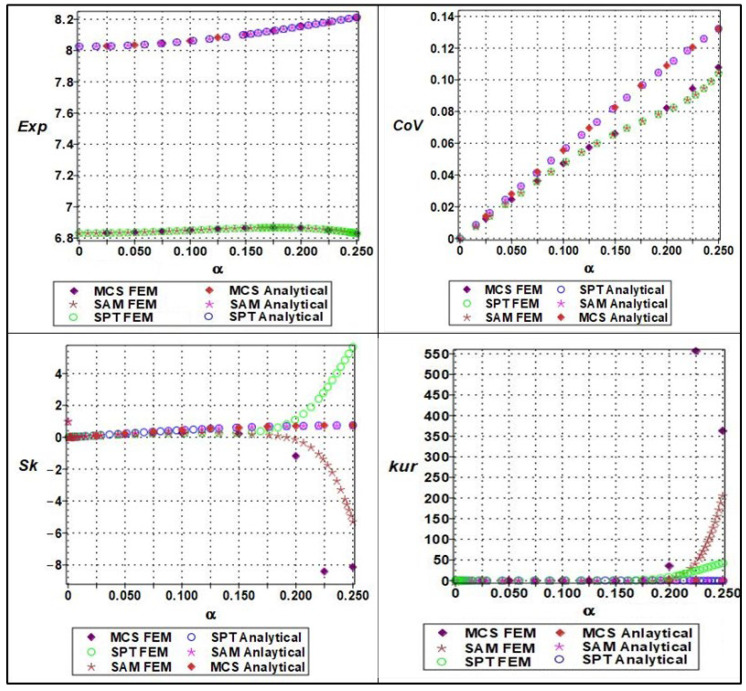
Statistical parameters (expectation, variation, skewness, and kurtosis) of analytical versus numerical of effective Young’s modulus with a series of material properties $\left(E_{m}\right)$.

The effective Young’s modulus of the cellular material exhibited a clear nonlinear response to increasing microstructural randomness, as captured by a sixth-order polynomial expansion concerning input variability. The randomness parameter α, which ranged from 0 to 0.25 in increments of 0.05, was applied to both material stiffness $(E_{m}),$ and geometric parameters such as cell length $\left(l_{s}\right)$. The expectation of the effective Young’s modulus showed a mild decreasing trend with increasing α, indicating that elevated randomness leads to a slight softening of the structure’s average stiffness, as shown in Figure 10.

This effect is attributable to the increased occurrence of weaker configurations within the stochastic ensemble. The coefficient of variation (COV), on the other hand, displayed a clearly nonlinear (near-quadratic) increase, confirming that even small input variability can lead to significant amplification in output variance. Higher-order statistical measures such as skewness and kurtosis also revealed the growing asymmetry and a peak of the modulus distribution with increasing randomness. Specifically, the skewness transitioned from near-zero to positive values, while kurtosis increased steadily, suggesting that the distribution becomes more right-tailed and sharply peaked. These effects were more accurately captured by Monte Carlo simulations and semi-analytical methods compared to stochastic perturbation techniques, which tended to underpredict higher moments at α>0.10. This highlights the importance of using comprehensive uncertainty quantification techniques when modeling effective stiffness in architected materials under manufacturing or material variability.

**Figure 11:**
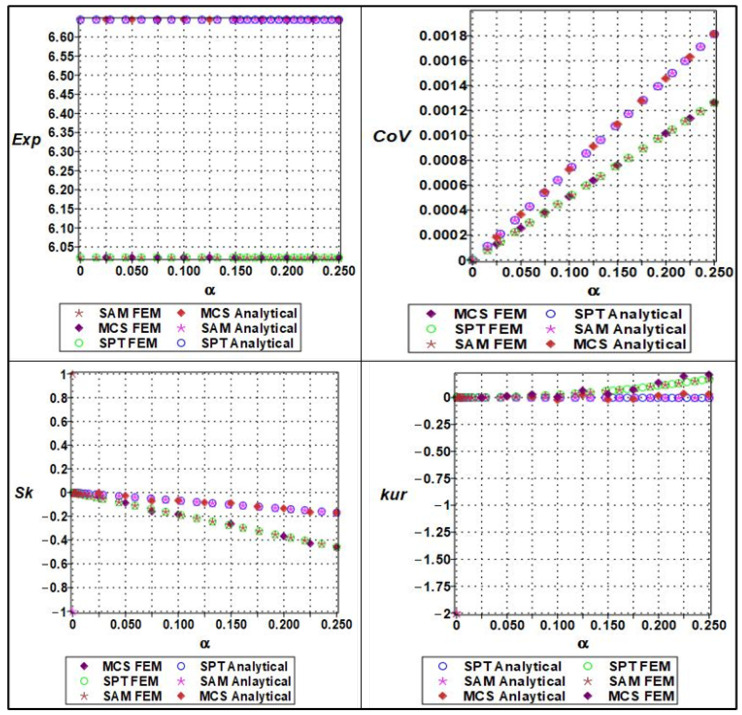
Statistical parameters (expectation, variation, skewness, and kurtosis) of analytical versus numerical of effective Young’s modulus with a series of lengths $\left(l_{s}\right)$.

The response of the effective yield strength to increasing input randomness exhibited significantly greater sensitivity compared to elastic stiffness. The yield strength was modeled as a sixth-order polynomial function of both cell length $\left(l_{s}\right)$ and wall thickness, $(t_{w}),$ with randomness parameterized by the coefficient of variation α. As α increased, the expectation of the yield strength declined sharply, especially when wall thickness variations dominated, due to the pronounced influence of local instabilities and stress concentration effects in thinner or uneven regions. The COV increased more rapidly than for Young’s modulus, exhibiting a pronounced nonlinear trend as shown in Figure 11. This reflects the inherently more unstable nature of plastic deformation, where localized yielding can initiate prematurely under geometrically perturbed conditions. Skewness values became markedly positive at higher levels of randomness, indicating a right-skewed distribution in which a significant number of realizations exhibited much lower yield strength than the mean. Similarly, kurtosis rose steeply with α, indicating a leptokurtic distribution with a heavy tail and an increased risk of extreme failure modes. The stochastic perturbation approach provided reasonable estimates for expectation and variance at low α, but failed to capture the nonlinear growth in skewness and kurtosis. In contrast, both Monte Carlo simulations and semi-analytical methods were able to track these changes effectively, making them essential tools for the reliable prediction of yield behavior in the presence of structural uncertainty.

**Figure 12:**
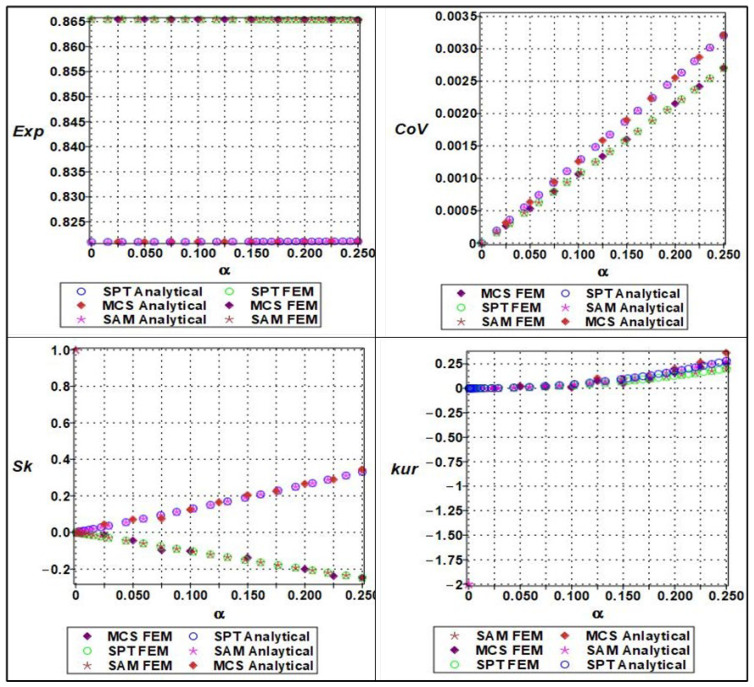
Statistical parameters (expectation, variation, skewness, and kurtosis) of analytical versus numerical of effective yield strength with a series of lengths $\left(l_{s}\right)$.

**Figure 13:**
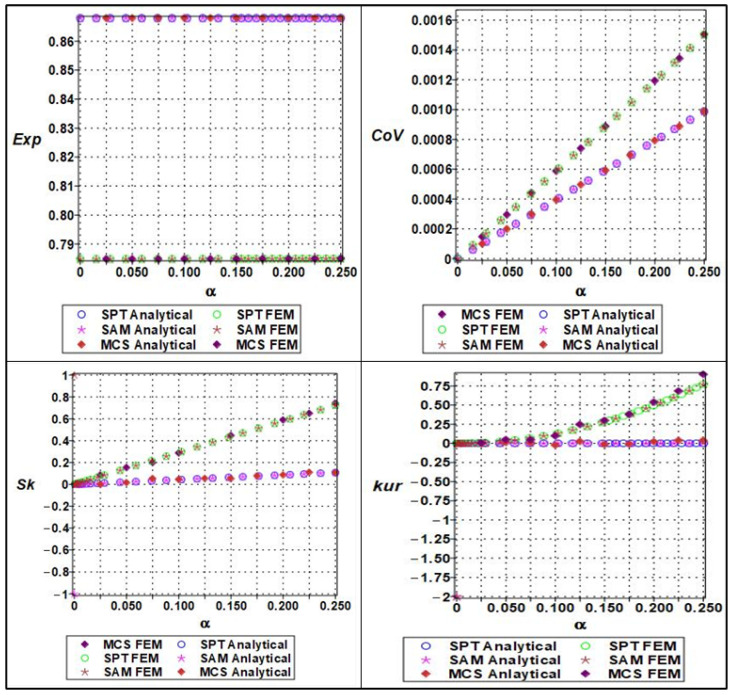
Statistical parameters (expectation, variation, skewness, and kurtosis) of analytical versus numerical of effective yield strength with a series of thicknesses of cell wall.

**Figure 14:**
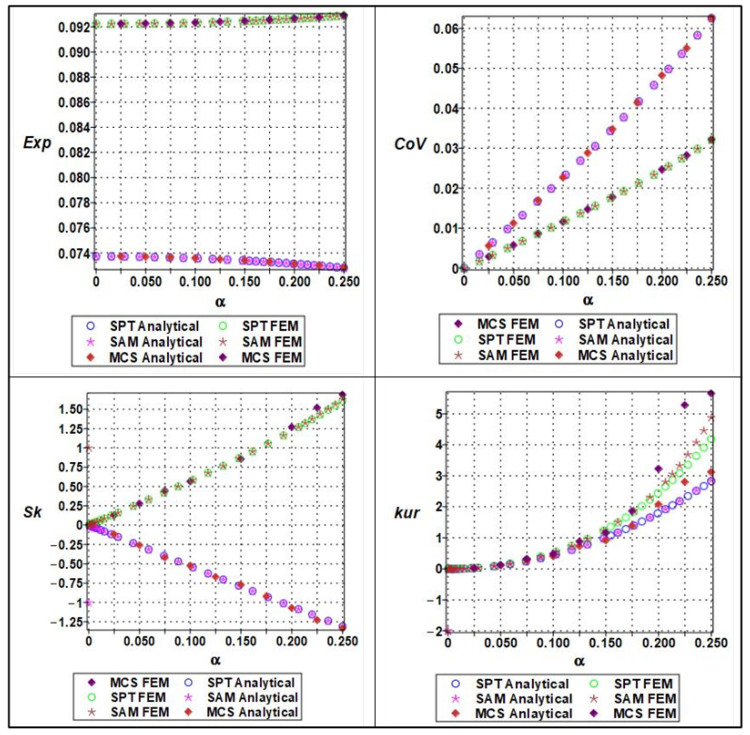
Statistical parameters (expectation, variation, skewness, and kurtosis) of analytical versus numerical of Poisson’s ratio with a series of length $\left(l_{s}\right)$.

**Figure 15:**
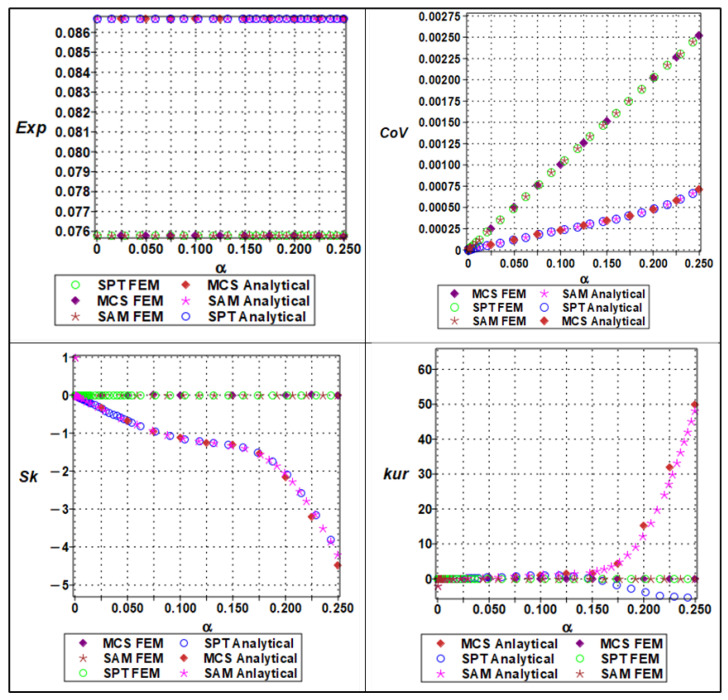
Statistical parameter (expectation, variation, skewness, and kurtosis) of analytical versus numerical of Poisson’s ratio with a series of thicknesses of cell wall $\left(t_{w}\right)$.

The effective Poisson’s ratio demonstrated a relatively muted response to increasing randomness compared to elastic modulus (seen in Figure 10 and Figure 11) and yield strength (seen in Figure 12 and Figure 13). Polynomial modeling of Poisson’s ratio under variability in cell length $\left(l_{s}\right)$ and wall thickness $\left(t_{w}\right)$ showed that the expectation remained nearly constant across the range of *α* as shown in Figure 14 and Figure 15 respectively, especially when cell length was the dominant uncertain parameter. This suggests that the global lateral-to-axial deformation behavior of the cellular material is less sensitive to small to moderate variations in microstructural geometry. The coefficient of variation increased slightly but linearly with *α*, implying a modest and predictable growth in dispersion. Skewness and kurtosis remained negligible for α≤0.10, only starting to rise marginally at higher levels of randomness, particularly when wall thickness was perturbed. These higher-order effects suggest that Poisson’s ratio, though generally stable, may exhibit some stochastic sensitivity when cell geometry becomes highly irregular. Unlike the cases of Young’s modulus and yield strength, stochastic perturbation techniques remained sufficiently accurate for all four statistical measures across the full range of α. This robustness, combined with the low sensitivity of Poisson’s ratio to geometric variability, makes it a relatively deterministic parameter in the mechanical characterization of cellular materials. Nevertheless, Monte Carlo and semi-analytical methods confirmed subtle nonlinear trends that may be critical in high-precision applications or when lateral compliance is a key design criterion.

The nonlinear response of the effective mechanical properties under uncertainty was modeled using sixth-order polynomial approximations derived from both analytical and numerical simulations. While this approach captures the essential trends in expectation, variance, skewness, and kurtosis of the output responses, the SPT exhibits limitations when applied beyond its rigorous domain of validity. In particular, SPT is mathematically reliable only for small perturbations, corresponding to low COV. In our study, the randomness parameter was varied up to COV = 0.275, and it was observed that SPT predictions begin to deviate at higher levels of input variability. Specifically, while SPT provides accurate estimates for the mean and variance at low to moderate COV (≤0.10–0.15), it systematically underpredicts skewness and kurtosis at larger perturbations, thereby failing to capture the emergence of heavy-tailed distributions and nonlinear amplification of fluctuations. MCS and SAM, by contrast, successfully reproduced these higher-order effects across the full range of variability. This comparative evidence demonstrates that SPT should be interpreted as reliable only in the low-COV regime while its application at higher variability levels is exploratory, with accuracy contingent upon benchmarking against more robust techniques such as MCS.

**Figure 16:**
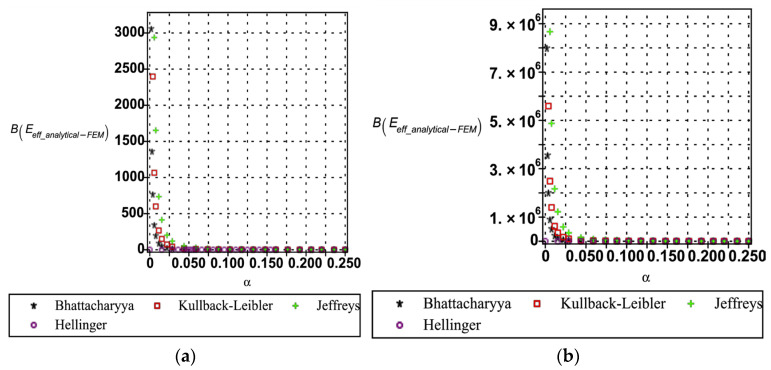
Relative entropy quantification of analytical and numerical Young’s modulus with respect to (**a**) based material $\left(E_{m}\right)$ and (**b**) segmental length $\left(l_{s}\right)$.

**Figure 17:**
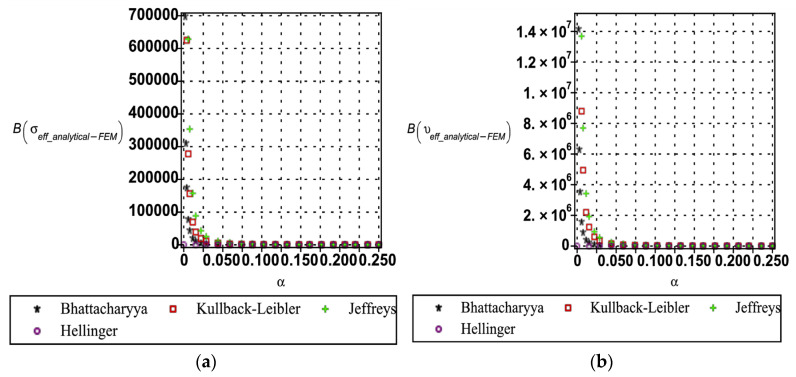
Relative entropy quantification of analytical and numerical yield strength with respect to variation in (**a**) segmental length $\left(l_{s}\right)$ and (**b**) cell wall thickness $\left(t_{w}\right)$.

**Figure 18:**
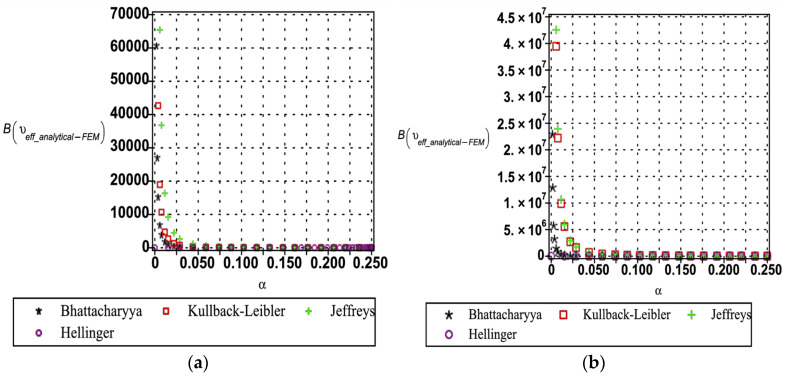
Relative entropy quantification of analytical and numerical Poisson’s ratio with respect to variation in (**a**) segmental length $\left(l_{s}\right)$ and (**b**) cell wall thickness $\left(t_{w}\right)$.

The entropy-based uncertainty quantification (as shown in Figure 16, Figure 17 and Figure 18) provides a rigorous framework for evaluating the divergence between analytical and numerical predictions of the effective mechanical properties of the hexagonal unit cell. Four relative entropy measures Kullback–Leibler, Jeffreys, Bhattacharyya, and Hellinger were employed to probe the sensitivity of Young’s modulus (see Figure 16), yield strength (see Figure 17), and Poisson’s ratio (cf. Figure 18) under perturbations in base material modulus $\left(E_{m}\right)$, segmental length $\left(l_{s}\right)$, and cell wall thickness $\left(t_{w}\right)$. The results demonstrate that Young’s modulus is the most sensitive property, with Kullback–Leibler, Jeffreys, and Bhattacharyya measures exhibiting sharp increases in divergence as perturbation magnitudes increase, reflecting asymmetry and heavy-tail amplification in the probability distributions. By contrast, the Hellinger distance remains comparatively small, indicating a stronger emphasis on distributional overlap and thereby highlighting regions of robustness. Yield strength displays moderate sensitivity, primarily governed by variations in wall thickness, while Poisson’s ratio shows consistently low divergence across all measures, signifying its stability under parametric uncertainty. Importantly, each entropy metric provides complementary insight: Kullback–Leibler and Jeffreys are most effective in capturing asymmetry and parameter-driven instabilities, Bhattacharyya is suited to quantify overall separability between distributions, and Hellinger highlights zones of stability. From an engineering perspective, these divergences can be interpreted as indicators of performance reliability. Low divergence values (<0.05 across KL/Jeffreys, <0.02 in Bhattacharyya/Hellinger) suggest negligible degradation, moderate values (0.05–0.2 in KL/Jeffreys, 0.02–0.1 in Bhattacharyya/Hellinger) point to diminishing reliability margins, and high divergences (>0.2 in KL/Jeffreys, >0.1 in Bhattacharyya/Hellinger) signal critical thresholds where predictive fidelity is compromised. Safety factors or design adjustments become necessary. This comparative interpretation establishes the practical utility of the entropy measures, linking statistical divergences directly to performance degradation in mechanical properties.

Overall, the comparison reveals that Bhattacharyya, KL, and Jeffreys relative entropies are more sensitive to deviations in tail behavior and skewness, while Hellinger offers more conservative, overlap-based divergence measures. This entropy-based analysis confirms that the accuracy of homogenization under uncertainty is highly property- and parameter-dependent, with Young’s modulus being the most prone to divergence and Poisson’s ratio the most robust.

## 7. Concluding Remarks

In this study, we determined the mechanical properties of 2D hexagonal cellular solids-Young’s modulus, yield strength, and Poisson’s ratio under uncertainty using Monte Carlo simulation (MCS), stochastic perturbation technique (SPT), and the semi-analytical method (SAM). Comparative results between analytical and FEM-based homogenization approaches show strong agreement in predicting expected values, with FEM providing superior accuracy in capturing localized nonlinear effects. The findings demonstrate that material modulus strongly influences stiffness-related properties, whereas geometric variations primarily affect deformation metrics. The coefficient of variation and higher-order statistics highlight the growing impact of randomness, particularly on Young’s modulus and yield strength, underscoring the necessity of probabilistic modeling for reliability in design. Among these methods, SPT is reliable only in the low-randomness regime, whereas SAM and MCS are required to capture the full distributional behavior at higher variability levels. Overall, the framework developed here provides a validated basis for uncertainty-aware design of the lightweight and multifunctional materials, with direct implications for biomedical, aerospace, and structural applications at various industrial levels.

## Figures and Tables

**Table 1:** Regression statistic of effective mechanical parameters of numerical and analytical results.

Regression Statistics		Multiple R	R Square	Adjusted R Square	Standard Error	Observations
Young’s Modulus w.r.t ($E_{m}$)	Numerically	0.999991	0.99998	0.999982	0.000575534	22
	Analytically	0.999991	0.99999	0.998982	7.20464 × 10^−16^	22
Young’s Modulus w.r.t ($l_{s}$)	Numerically	0.999614	0.99923	0.999190	0.005915965	22
	Analytically	0.999958	0.99992	0.999912	0.00233744	22
Yield strength w.r.t ($l_{s}$)	Numerically	0.999958	0.99992	0.999912	0.00233744	22
	Analytically	0.999008	0.99802	0.997918	0.001220271	22
Yield strength w.r.t ($t_{w}$)	Numerically	0.993348	0.98674	0.986077	0.006089778	22
	Analytically	0.999066	0.99813	0.998040	0.002416551	22
Poisson’s ratio w.r.t ($l_{s}$)	Numerically	0.931125	0.86699	0.852216	0.001243435	11
	Analytically	0.999831	0.99966	0.999625	3.30709 × 10^−5^	11
Poisson’s ratio w.r.t ($t_{w}$)	Numerically	0.929785	0.8645	0.849444	0.000729089	11
	Analytically	0.935685	0.87256	0.978552	5.25507 × 10^−18^	11

## Data Availability

The original contributions presented in this study are included in the article. Further inquiries can be directed to the corresponding author.

## Appendix A

**Table A1:** FEM mesh details for determination of Young’s modulus.

Model Description		Elastic Model					
		Variation on (*E*_*m*_)			Variation on (*l*_*s*_)		
		Mesh		Nodes	Mesh		Nodes
		Elements	Types	No.	Elements	Types	No.
Case-1	Case-1-8N-Q	756	CPS8R	2498	932	CPS8R	3026
	Case-1-8N-QD	726 14	CPS8R CPS6M	2436	897 34	CPS8R CPS6M	2989
Case-2	Case-2-8N-Q	756	CPS8R	2498	780	CPS8R	2570
	Case-2-8N-QD	726 14	CPS8R CPS6M	2436	757 18	CPS8R CPS6M	2537
Case-3	Case-3-8N-Q	756	CPS8R	2498	771	CPS8R	2543
	Case-3-8N-QD	726 14	CPS8R CPS6M	2436	736 20	CPS8R CPS6M	2478
Case-4	Case-4-8N-Q	756	CPS8R	2498	747	CPS8R	2471
	Case-4-8N-QD	726 14	CPS8R CPS6M	2436	721 14	CPS8R CPS6M	2421
Case-5	Case-5-8N-Q	756	CPS8R	2498	741	CPS8R	2453
	Case-5-8N-QD	726 14	CPS8R CPS6M	2436	704 20	CPS8R CPS6M	2382
Case-6	Case-6-8N-Q	756	CPS8R	2498	741	CPS8R	2453
	Case-6-8N-QD	726 14	CPS8R CPS6M	2436	706 10	CPS8R CPS6M	2368
Case-7	Case-7-8N-Q	756	CPS8R	2498	698	CPS8R	2324
	Case-7-8N-QD	726 14	CPS8R CPS6M	2436	666 20	CPS8R CPS6M	2268
Case-8	Case-8-8N-Q	756	CPS8R	2498	706	CPS8R	2348
	Case-8-8N-QD	726 14	CPS8R CPS6M	2436	656 26	CPS8R CPS6M	2250
Case-9	Case-9-8N-Q	756	CPS8R	2498	704	CPS8R	821
	Case-9-8N-QD	726 14	CPS8R CPS6M	2436	674 16	CPS8R CPS6M	2288
Case-10	Case-10-8N-Q	756	CPS8R	2498	705	CPS8R	2351
	Case-10-8N-QD	726 14	CPS8R CPS6M	2436	668 16	CPS8R CPS6M	2272
Case-11	Case-11-8N-Q	756	CPS8R	2498	703	CPS8R	2345
	Case-11-8N-QD	726 14	CPS8R CPS6M	2436	666 24	CPS8R CPS6M	2282

**Table A2:** FEM mesh details for determination of yield strength.

Model Description		Plastic Model					
		Variation on (*t*_*w*_)			Variation on (*l*_*s*_)		
		Mesh		Nodes	Mesh		Nodes
		Elements	Types	No.	Elements	Types	No.
Case-1	Case-1-8N-Q	421	CPS8R	1467	413	CPS8R	1443
	Case-1-8N-QD	394 20	CPS8R CPS6M	1426	408 4	CPS8R CPS6M	1436
Case-2	Case-2-8N-Q	423	CPS8R	1473	436	CPS8R	1512
	Case-2-8N-QD	140 12	CPS8R CPS6M	1458	417 12	CPS8R CPS6M	1479
Case-3	Case-3-8N-Q	417	CPS8R	1455	418	CPS8R	1458
	Case-3-8N-QD	403 14	CPS8R CPS6M	1441	402 10	CPS8R CPS6M	1430
Case-4	Case-4-8N-Q	416	CPS8R	1452	441	CPS8R	1527
	Case-4-8N-QD	387 14	CPS8R CPS6M	1393	420 12	CPS8R CPS6M	1488
Case-5	Case-5-8N-Q	408	CPS8R	1428	407	CPS8R	1425
	Case-5-8N-QD	386 20	CPS8R CPS6M	1402	386 16	CPS8R CPS6M	1394
Case-6	Case-6-8N-Q	414	CPS8R	1446	423	CPS8R	1473
	Case-6-8N-QD	391 14	CPS8R CPS6M	1405	396 12	CPS8R CPS6M	1416
Case-7	Case-7-8N-Q	420	CPS8R	1464	408	CPS8R	1428
	Case-7-8N-QD	404 8	CPS8R CPS6M	1432	388 12	CPS8R CPS6M	1392
Case-8	Case-8-8N-Q	446	CPS8R	1542	441	CPS8R	1533
	Case-8-8N-QD	413 12	CPS8R CPS6M	1467	415 12	CPS8R CPS6M	1479
Case-9	Case-9-8N-Q	422	CPS8R	1470	445	CPS8R	1547
	Case-9-8N-QD	402 22	CPS8R CPS6M	515	419 10	CPS8R CPS6M	1489
Case-10	Case-10-8N-Q	425	CPS8R	1479	451	CPS8R	1569
	Case-10-8N-QD	401 12	CPS8R CPS6M	1431	439 6	CPS8R CPS6M	1545
Case-11	Case-11-8N-Q	418	CPS8R	1458	448	CPS8R	1560
	Case-11-8N-QD	408 14	CPS8R CPS6M	1456	440 6	CPS8R CPS6M	1548

**Table A3:** FEM mesh details for determination of Poisson’s ratio.

Model Description		Poisson’s Ratio Model					
		Variation on (*t*_*w*_)			Variation on (*l*_*s*_)		
		Mesh		Nodes	Mesh		Nodes
		Elements	Types	No.	Elements	Types	No.
Case-1	Case-1-3N-T	796	CPS3	500	816	CPS3	510
Case-2	Case-2-3N-T	804	CPS3	504	812	CPS3	508
Case-3	Case-3-3N-T	804	CPS3	504	812	CPS3	508
Case-4	Case-4-3N-T	812	CPS3	508	810	CPS3	507
Case-5	Case-5-3N-T	812	CPS3	508	812	CPS3	508
Case-6	Case-6-3N-T	816	CPS3	510	810	CPS3	507
Case-7	Case-7-3N-T	812	CPS3	508	812	CPS3	508
Case-8	Case-8-3N-T	818	CPS3	511	840	CPS3	525
Case-9	Case-9-3N-T	810	CPS3	507	842	CPS3	526
Case-10	Case-10-3N-T	810	CPS3	507	868	CPS3	542
Case-11	Case-11-3N-T	812	CPS3	508	872	CPS3	544

## Appendix B

Regression analysis of the effective mechanical parameters w.r.t changing variable (material property ($E_{m}$), segmental length ($l_{s}$), and thickness of cell wall ($t_{w}$)).

**Effective Parameters**			**Standard Error**	**t Stat**	***p*-Value**	**Lower 95%**	**Upper 95%**	**Lower 99.0%**	**Upper 99.0%**
Young’s Modulus w.r.t ($E_{m}$)	Numerically	Intercept	0.005356153	9.92399 × 10^−5^	0.059921801	−0.01117	0.011173271	−0.015239543	0.015240606
		$E_{m}$	7.7605 × 10^−5^	1080.210138	3.85534 × 10^−49^	0.083668	0.083991624	0.08360893	0.084050556
	Analytically	Intercept	6.70493 × 10^−15^	192198474.2	3.8133 × 10^−154^	1.29 × 10^−6^	1.28868 × 10^−6^	1.28868 × 10^−6^	1.28868 × 10^−6^
		$E_{m}$	9.71473 × 10^−17^	9.63963 × 10^+14^	3.7592 × 10^−288^	0.093646	0.093646399	0.093646399	0.093646399
Young’s Modulus w.r.t ($l_{s}$)	Numerically	Intercept	0.060638964	255.1613197	1.3144 × 10^−36^	15.34623	15.59920885	15.30017973	15.64525664
		$l_{s}$	0.013295142	−160.961054	1.31427 × 10^−32^	−2.16773	−2.11226682	−2.177829194	−2.10217081
	Analytically	Intercept	0.023958893	750.4155359	5.6249 × 10^−46^	17.92915	18.02910283	17.91095427	18.04729664
		$l_{s}$	0.005253006	−487.911067	3.08416 × 10^−42^	−2.57396	−2.55204242	−2.577946588	−2.54805341
Yield strength w.r.t ($l_{s}$)	Numerically	Intercept	0.013154723	172.8301002	3.17056 × 10^−33^	2.246092	2.300972401	2.236102473	2.310961786
		$l_{s}$	0.002884184	−110.282053	2.50836 × 10^−29^	−0.32409	−0.31205739	−0.326280172	−0.30986721
	Analytically	Intercept	0.012507849	163.1960621	9.97684 × 10^−33^	2.015141	2.06732257	2.005642576	2.076820734
		$l_{s}$	0.002742356	−100.333317	1.65641 × 10_−28_	−0.28087	−0.26942921	−0.282952595	−0.26734673
Yield strength w.r.t ($t_{w}$)	Numerically	Intercept	0.044771257	−19.2876476	2.15579 × 10^−14^	−0.95692	−0.77014103	−0.99092167	−0.7361428
		$t_{w}$	0.041057273	38.57814888	2.97942 × 10^−20^	1.49827	1.669557567	1.467091706	1.700735486
	Analytically	Intercept	0.017766168	−49.962198	1.77986 × 10^−22^	−0.9247	−0.85057723	−0.938187591	−0.83708602
		$t_{w}$	0.016292382	103.426271	9.0352 × 10^−29^	1.651075	1.719045601	1.638702928	1.731417649
Poisson’s ratio w.r.t ($l_{s}$)	Numerically	Intercept	0.018024544	12.44637904	5.63782 × 10^−7^	0.183566	0.265114651	0.165763498	0.282917103
		ls	0.003951896	−7.65939614	3.12819 × 10^−5^	−0.03921	−0.02132933	−0.043112145	−0.01742612
	Analytically	Intercept	0.000479388	326.2699766	1.21486 × 10^−19^	0.155325	0.157494365	0.154851982	0.157967846
		$l_{s}$	0.000105106	−163.197846	6.19214 × 10^−17^	−0.01739	−0.01691534	−0.017494684	−0.01681153
Poisson’s ratio w.r.t ($t_{w}$)	Numerically	Intercept	0.00758042	4.125197252	0.002577881	0.014123	0.048418826	0.006635609	0.055905843
		$t_{w}$	0.006951588	7.577620388	3.405 × 10^−5^	0.036951	0.068402084	0.030084979	0.075268018
	Analytically	Intercept	5.46375 × 10^−17^	0.253997414	0.805205267	−1.1 × 10^−16^	1.37476 × 10^−16^	−1.63685 × 10^−16^	1.91441 × 10^−16^
		$t_{w}$	5.01051 × 10^−17^	1.4539 × 10^+15^	1.7541 × 10^−133^	0.072848	0.072847682	0.072847682	0.072847682
